# PbrSLAH3 is a nitrate-selective anion channel which is modulated by calcium-dependent protein kinase 32 in pear

**DOI:** 10.1186/s12870-019-1813-z

**Published:** 2019-05-08

**Authors:** Guodong Chen, Li Wang, Qian Chen, Kaijie Qi, Hao Yin, Peng Cao, Chao Tang, Xiao Wu, Shaoling Zhang, Peng Wang, Juyou Wu

**Affiliations:** Center of Pear Engineering Technology Research, State Key Laboratory of Crop Genetics and Germplasm Enhancement, College of Horticulture, Nanjing Agricultural University, No 6. Tongwei Road, Nanjing, China

**Keywords:** Nitrogen, Signaling, Anion channel, Nitrate, PbrSLAH3, Phosphorylation

## Abstract

**Background:**

The functional characteristics of *SLAC/SLAH* family members isolated from *Arabidopsis thaliana*, poplar, barley and rice have been comprehensively investigated. However, there are no reports regarding *SLAC/SLAH* family genes from *Rosaceae* plants*.*

**Results:**

In this study, the function of PbrSLAH3, which is predominately expressed in pear (*Pyrus bretschneideri*) root, was investigated. PbrSLAH3 can rescue the ammonium toxicity phenomenon of *slah3* mutant plants under high-ammonium/low-nitrate conditions. In addition, yeast two-hybrid and bimolecular fluorescence complementation assays confirmed that PbrSLAH3 interacts with PbrCPK32. Moreover, when *PbrSLAH3* was co-expressed with either the *Arabidopsis* calcium-dependent protein kinase (*CPK*) *21* or *PbrCPK32* in *Xenopus* oocytes, yellow fluorescence was emitted from the oocytes and typical anion currents were recorded in the presence of extracellular NO_3_^−^. However, when *PbrSLAH3* alone was injected, no yellow fluorescence or anion currents were recorded, suggesting that anion channel PbrSLAH3 activity was controlled through phosphorylation. Finally, electrophysiological and transgene results showed that PbrSLAH3 was more permeable to NO_3_^−^ than Cl^−^.

**Conclusion:**

We suggest that PbrSLAH3 crossing-talk with PbrCPK32 probably participate in transporting of nitrate nutrition in pear root.

**Electronic supplementary material:**

The online version of this article (10.1186/s12870-019-1813-z) contains supplementary material, which is available to authorized users.

## Background

Anion channels, which widely exist in a variety of bio-membranes, play an important role in cell signaling, osmoregulation, metabolism, stress tolerance and plant nutrition [1–4]. Electrophysiological data from different research groups confirmed that rapid-type (R-type) and slow-type (S-type) anion channels are involved in anion transport [5–7]. By far, *SLAC/SLAH* family genes (S-type anion channels) from species, including *Arabidopsis thaliana*, poplar, barley and rice, have been identified and studied, five members in *Arabidopsis* [8, 9], seven members in *Populus* [10] and nine members in rice [11]. However, no reports regarding *SLAC/SLAH* family genes from *Rosaceae* plants have been found.

To date, only the physiological functions of the *SLAC/SLAH* family members in *Arabidopsis* have been widely studied [12–15]. *AtSLAC1*, together with *SLAC1* homologous 3 (*AtSLAH3*), plays an important role in regulating ABA-induced stomatal closure [16–19]. During this process, ABA is firstly perceived by the RCAR/PYR/PYL–ABI1 complex, which then releases protein kinase from *ABI1*-induced inhibition, and then AtSLAC1 and AtSLAH3 are activated by the calcium-independent protein kinase OST1 and the calcium-dependent protein kinases (CPKs) of *Arabidopsis* [13]. Meanwhile, other members of the *SLAC/SLAH* family in *Arabidopsis* have also been intensively studied. The results from the Geiger group showed that SLAH1, as a silent subunit, facilitates SLAH3-mediated chloride efflux from pericycle cells into the root xylem vessels [20, 21]. When co-expression the silent *SLAH1* subunit and *SLAH3* in *Xenopus* oocytes, SLAH3 was activated even in the absence of nitrate and calcium-dependent protein kinases [20]. Besides, *SLAH2*, another member of the *SLAC/SLAH* family, which is expressed in the stele of the root, acts in a nitrate-specific channel that is impermeable to chloride when co-expressed with protein kinases in *Xenopus* oocytes [22].

Although AtSLAC1 and AtSLAH3 exhibit similar physiological functions in guard cells, different expression patterns and functional characteristics were observed between these two channels. For instance, *AtSLAC1* is exclusively expressed in the guard cells [23], while *AtSLAH3*, in addition to being expressed in the guard cells, is also predominantly expressed in the root and weakly expressed in the pollen tube [24, 25]. Furthermore, AtSLAC1 exhibits a high permeability to NO_3_^−^ than Cl^−^, with a P(NO_3_^−^)/P(Cl^−^) ratio of 10 [22], while AtSLAH3 is a nitrate-activated S-type anion channel with a P(NO_3_^−^)/P(Cl^−^) ratio of 20 [13, 14]. Apart from regulating the stomatal closure together with *AtSLAC1*, *AtSLAH3* alleviates ammonium toxicity and regulates pollen tube growth in *Arabidopsis* [25].

More importantly*, SLAC/SLAH* family genes from different plant species with high similarity showed varied expression patterns and functional characteristics. For instance, in contrast to AtSLAC1, which is permeable to both chloride and nitrate, OsSLAC1 is identified as a nitrate-selective anion channel [11]. Furthermore, in contrast to *AtSLAH3*, which is predominantly expressed in the root and is dependent on phosphorylation activation, *PttSLAH3*, which is predominantly expressed in poplar secretory epithelia, is independent of activation by protein kinases [10]. Thus, based on these results mentioned above, we speculated that *SLAH3* isolated from the pear, which has not been clarified until recently, may own special functional-characteristics.

Nitrate is the major source of nitrogen for plants in nature and in agriculture under aerobic conditions [14, 26, 27]. *SLAC/SLAH* genes from different species have been proven to play an important role in nitrate transport. In this study, we focused on investigating the function of *PbrSLAH3*, which is predominantly expressed in pear root verified by RT-PCR, qRT-PCR and *β*-Glucuronidase (GUS) staining assays. The physiological function of *PbrSLAH3* was investigated by its over-expression in a *slah3* mutant. In consistent with that of *AtSLAH3*, *PbrSLAH3* also participates in alleviation of ammonium toxicity under high ammonium/low nitrate conditions. Furthermore, both yeast two-hybrid and bimolecular fluorescence complementation (BiFC) assays confirmed the interaction between PbrSLAH3 and PbrCPK32. Finally, an electrophysiological experiment revealed that PbrSLAH3 is activated by PbrCPK32 and the channel is highly selective for nitrate without obvious permeability to chloride. Thus, we conclude that PbrSLAH3 serve as a nitrate efflux channel and may participate in nitrate transport in pear root.

## Results

### The phylogeny and expression patterns of *SLAC/SLAH* genes in pear

**Fig. 1:**
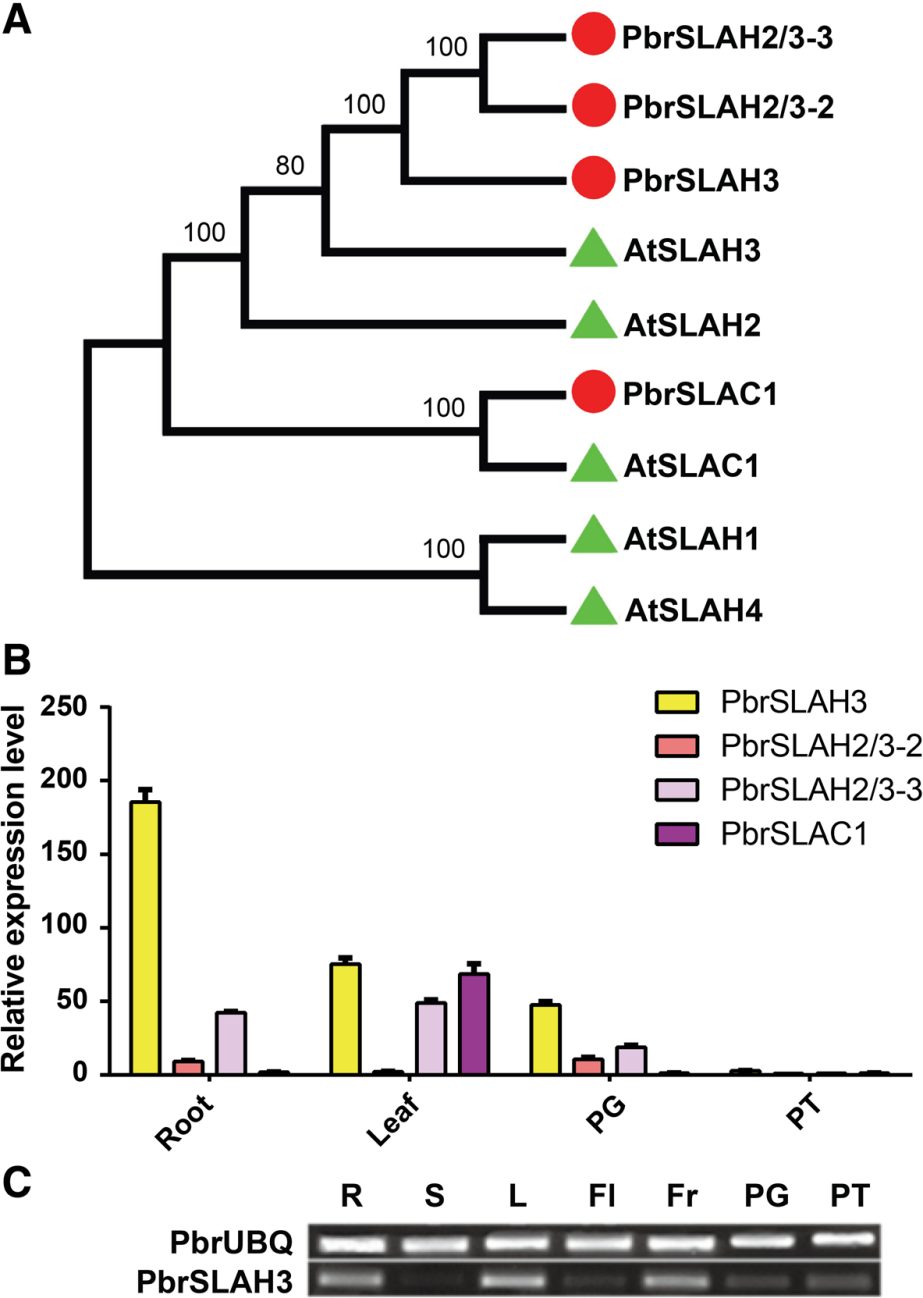
The determination of S-type anion channels in pear and the expression analysis. **a** Phylogenetic tree of the *Arabidopsis thaliana* SLAC/SLAH anion channel family and the pear orthologs. Four putative PbrSLAC/SLAH anion channels were identified in the *Pyrus* genome database. *PbrSLAC1* appears to be related to *AtSLAC1*, other three homologs were classified into the *AtSLAH2/3* group, while *AtSLAH1/4* homologs could not be identified in pear. The *PbrSLAH2/3–1* sequence was used to clone the ortholog from *Pyrus bretschneideri* and named *PbrSLAH3*. **b** The expression patterns of *PbrSLAC/SLAH* family genes in the root, leaf, pollen grain and pollen tube of pear were analyzed by qRT-PCR. The values displayed in the figure were the geometric means of the relative expression of *PbrSLAC/SLAH* when both *PbrTUB* and *PbrUBQ* as reference genes. Three biological and three technical replicates were processed for the qRT-PCR assays. The error bars indicate standard deviations. The data are shown as mean values ± SDs. **c** RT-PCR was used to confirm the expression patterns of the *PbrSLAH3* gene and *PbrUBQ* gene was used as an internal control

*AtSLAC/SLAH* orthologous in pear were identified by a BLASTp algorithm-based search of the pear genomic database (http://peargenome.njau.edu.cn/) using the amino acid sequences of AtSLAC/SLAH as queries. To compare the similarity levels between the *PbrSLAC/SLAH* and *AtSLAC/SLAH*, a rooted phylogenetic tree was constructed using the neighbor-joining method (Fig. 1a). As shown in Fig. 1, PbrSLAC1 and AtSLAC1 were grouped to the same branch, while other three PbrSLAC/SLAH proteins, together with AtSLAH2 and AtSLAH3, belonged to another branch. Orthologs of AtSLAC/SLAH in pear did not phylogenetically cluster with AtSLAH1 and AtSLAH4. To further determine which genes in the *PbrSLAC/SLAH* family may participate in nitrate transporting in pear root, the expression patterns of *PbrSLAC/SLAH* family genes in the root, leaf, pollen grain and pollen tube were analyzed by quantitative and semi-quantitative RT-PCR (Fig. 1b and c). *PbrSLAC1* was exclusively expressed in leaf, while *PbrSLAH3*, *PbrSLAH2/3–2* and *PbrSLAH2/3–3* were widely expressed in root, leaf and pollen. *PbrSLAH3* displayed a relative higher expression level than the other *PbrSLAH2/3* genes in pear root (Fig. 1b). Furthermore, the transcriptome data analysis suggested that the expression level of *PbrSLAH3* was up-regulated during nitrate starvation and then, after the re-supply of nitrate, its expression level was gradually down-regulated (Additional file 1: Figure S1).Taken together these results, we presume that *PbrSLAH3* may be involved in nitrate transport in pear root and may play an important role in nitrate nutrition in pear trees.

### Subcellular localization and accurate organizational positions of *PbrSLAH3*

**Fig. 2:**
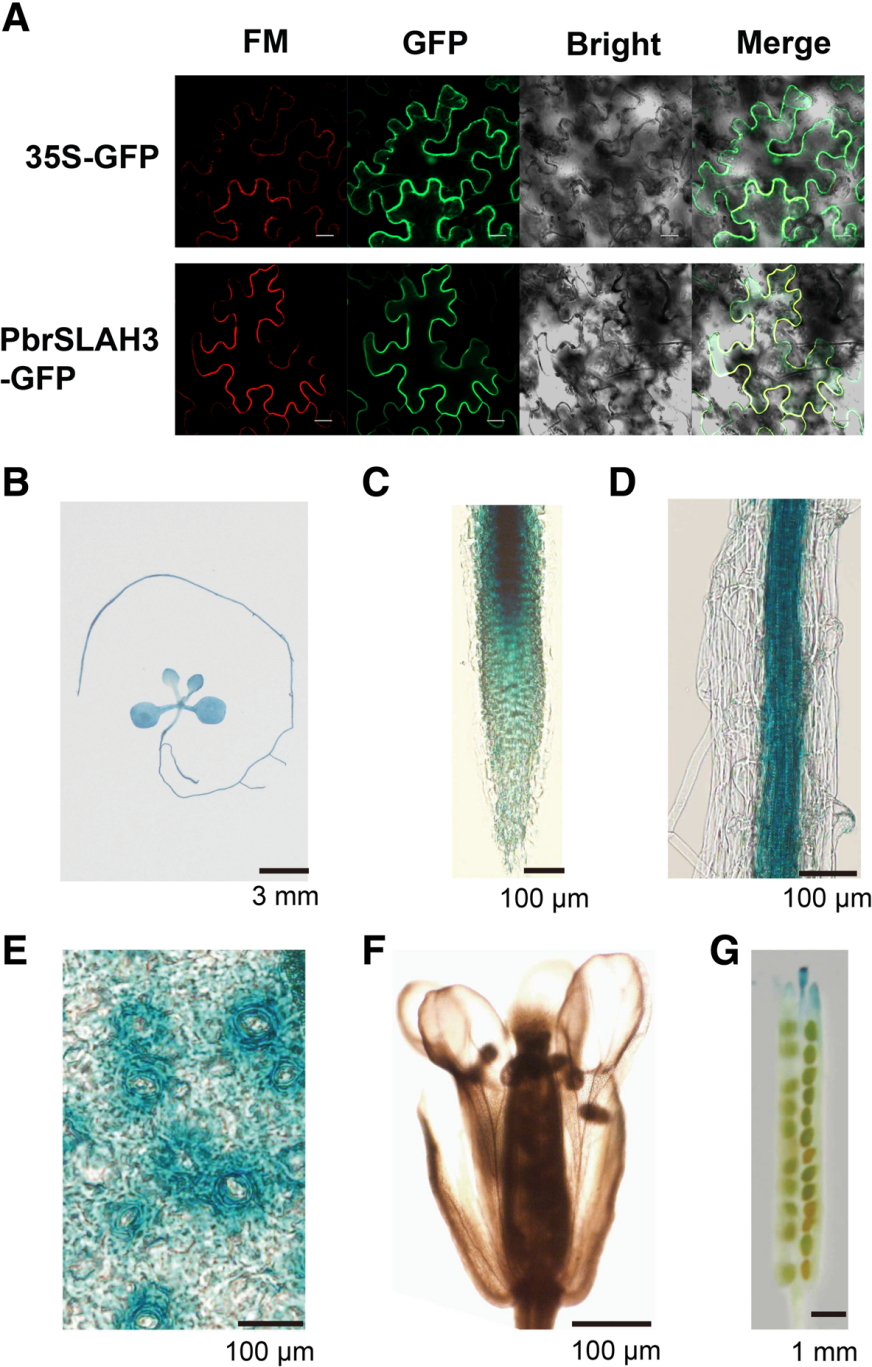
The subcellular localization of *PbrSLAH3* to the plasma membrane, and a histochemical analysis of GUS activity in *PbrSLAH3* transgenic *Arabidopsis* (Col-0) plants. **a** GFP fluorescence emitted from the leaves of tobacco infected by *Agrobacterium* harboring *PbrSLAH3*-GFP was detected under a confocal microscope and the plasma membranes are stained red by FM4–64. GFP: green fluorescent protein; Chl: chlorophyll; Bright: bright-field image of *Agrobacterium tumefaciens*-infiltrated tobacco leaves; Merge: merged fluorescent images; Bar = 10 μm. **b**–**g** GUS-histochemical analysis performed with *Arabidopsis* plants expressing the reporter gene under the control of the *PbrSLAH3* promoter. *PbrSLAH3* was expressed in young leaves, mature leaves, roots, stems, epidermal hairs, stomatal and siliques

To determine the subcellular localization of *PbrSLAH3*, the fusion gene of *PbrSLAH3*-GFP was constructed and transformed into tobacco (*N. benthamiana*) leaves. As previously reported, the control GFP was uniformly distributed throughout the whole cell, and the PbrSLAH3-GFP fusion protein was observed exclusively in the plasma membrane with a Zeiss LSM 780 Image Browser (Fig. 2a). To further identify the accurate expression profiles of *PbrSLAH3* in pear, transgenic *Arabidopsis* plants carrying a GUS gene under control of the *PbrSLAH3* promoter fragment (2000 bp) were generated. The GUS activity assays showed that *PbrSLAH3* is strongly expressed in roots, young leaves, mature leaves and stoma, and is weakly expressed in stems. However, it is not expressed in seed and silique (Fig. 2b-g). Thus, we presume that *PbrSLAH3* may have diverse functions similar to those of *AtSLAH3*, which plays an important role in the alleviation of ammonium toxicity and regulation of stomatal closure and pollen growth.

### Functional characterization of *PbrSLAH3* in *Arabidopsis*

**Fig. 3:**
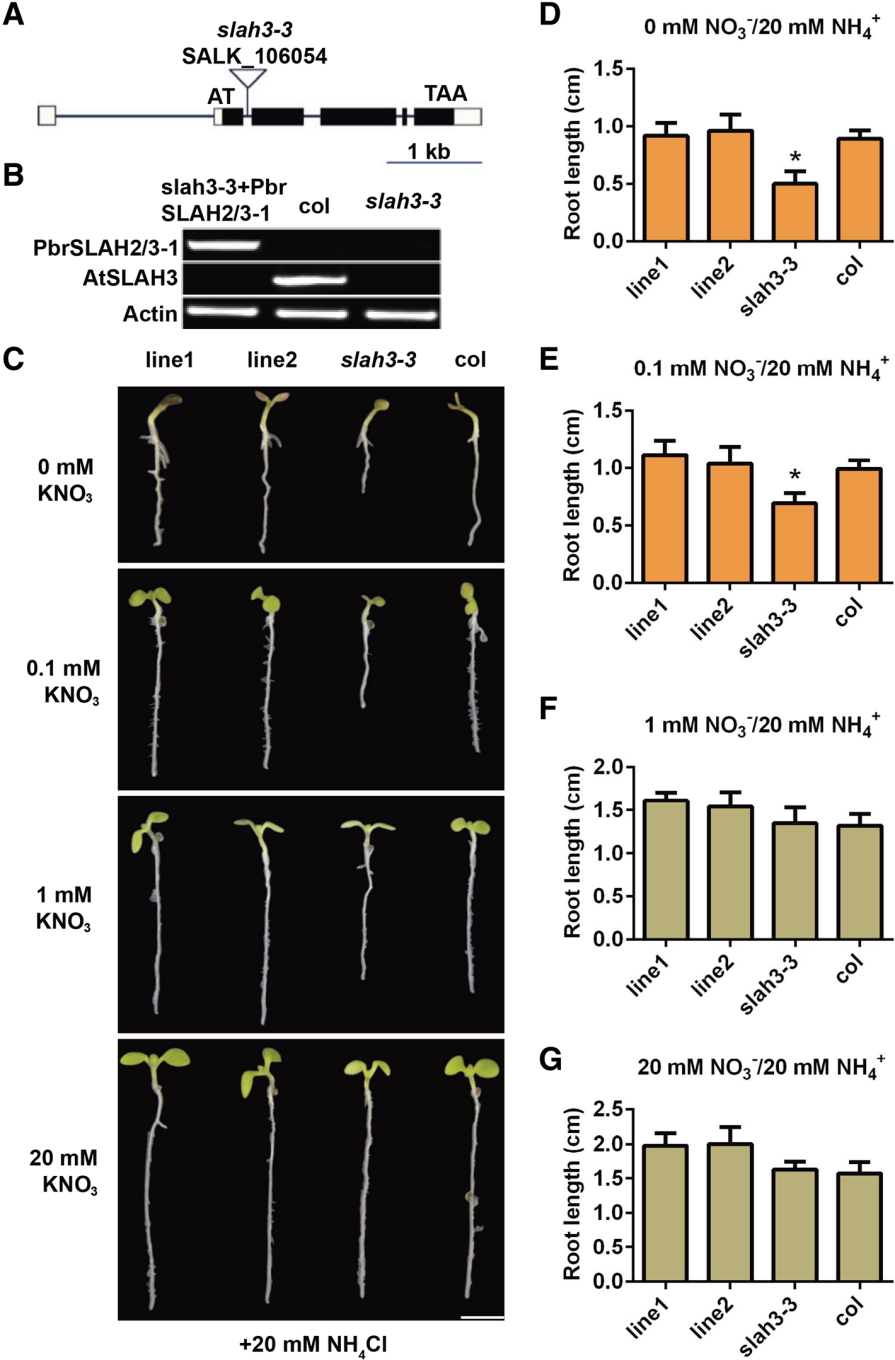
PbrSLAH3 rescues the ammonium toxicity of *slah3–3* mutant plants under high ammonium/low-nitrate conditions. **a** Schematic map of the T-DNA insertion site in the slah3–3 mutant. **b** RT-PCR analyses of *PbrSLAH3* and *AtSLAH3* expression levels in transgenic, wild-type (Col-0) and slah3–3 mutant plants. Actin was used as a control. **c** Growth comparison among 7-d-old transgenic lines 1 and 2, wild-type (Col-0) and slah3–3 mutant seedlings grown on 1/2 N-free Murashige and Skoog medium supplemented with different concentrations of KNO_3_ (0–20 mM) and 20 mM NH_4_Cl. **d**–**g** The average primary root lengths of seedlings under the same conditions as displayed in (**c**) were statistical analyzed. Results are averages ± SEs of three independent experiments (*n* = 15 per experiment). The asterisk represents statistical significance among transgenic lines, wild-type and slah3–3 mutant plant (Student’s t-test, *P < 0.05). Bar = 0.5 cm

In line with previous reports, the *Arabidopsis slah3–3* mutant exhibited more severe ammonium toxicity than wild-type when grown on plates containing a high-ammonium/low-nitrate medium [25]. As shown in Fig. 3a, the T-DNA insertion site in the slah3–3 mutant was in the second intron of *AtSLAH3*. To examine the physiological function of *PbrSLAH3*, complementation lines of the *Arabidopsis slah3–3* mutant were constructed by the gene of *PbrSLAH3*. Two transgenic lines were selected based on the RT-PCR results. The mRNA of *PbrSLAH3* was not detected in wild-type and *slah3–3* mutant plants, but it was highly expressed in the transgenic lines (Fig. 3b). Phenotype testing showed that the ammonium toxicity phenotype of the *slah3–3* mutant under high-ammonium/low-nitrate conditions was rescued by the overexpression of *PbrSLAH3* (Fig. 3c and Additional file 1: Figure S2). Statistical results demonstrated that the root length of the *slah3–3* mutant was approximately 56–70% that of the wild-type when grown on high NH_4_^+^ medium supplied with 0 or 0.1 mM KNO_3_, while there were no differences between each of the complementation lines and the wild-type (Fig. 3d–g), suggesting that *PbrSLAH3* plays an important role in the alleviation of ammonium toxicity under nitrate-limited conditions. Furthermore, when the KNO_3_ concentration in the medium reaching up to 1 mM or more (20 mM), no clear differences were observed between the *slah3–3* mutant and wild-type, indicating that sufficient levels of nitrate can counteract ammonium toxicity.

### Identification of *PbrSLAH3* regulators

Considering the activation of the AtSLAH3*-*encoded channel requires the protein kinase AtCPK21 [10, 13], and *PbrSLAH3* shows high similarity and similar function to that of *AtSLAH3*, we investigated whether PbrSLAH3 can interact with AtCPK21 to transport nitrate ions. BiFC assays showed that PbrSLAH3 can interact with AtCPK21 in the plasma membrane of tobacco epidermal cells (Additional file 1: Figure S3), indicating that PbrSLAH3 may be involved in a signal-transduction pathway through phosphorylation and it may be a counterpart of AtSLAH3.

**Fig. 4:**
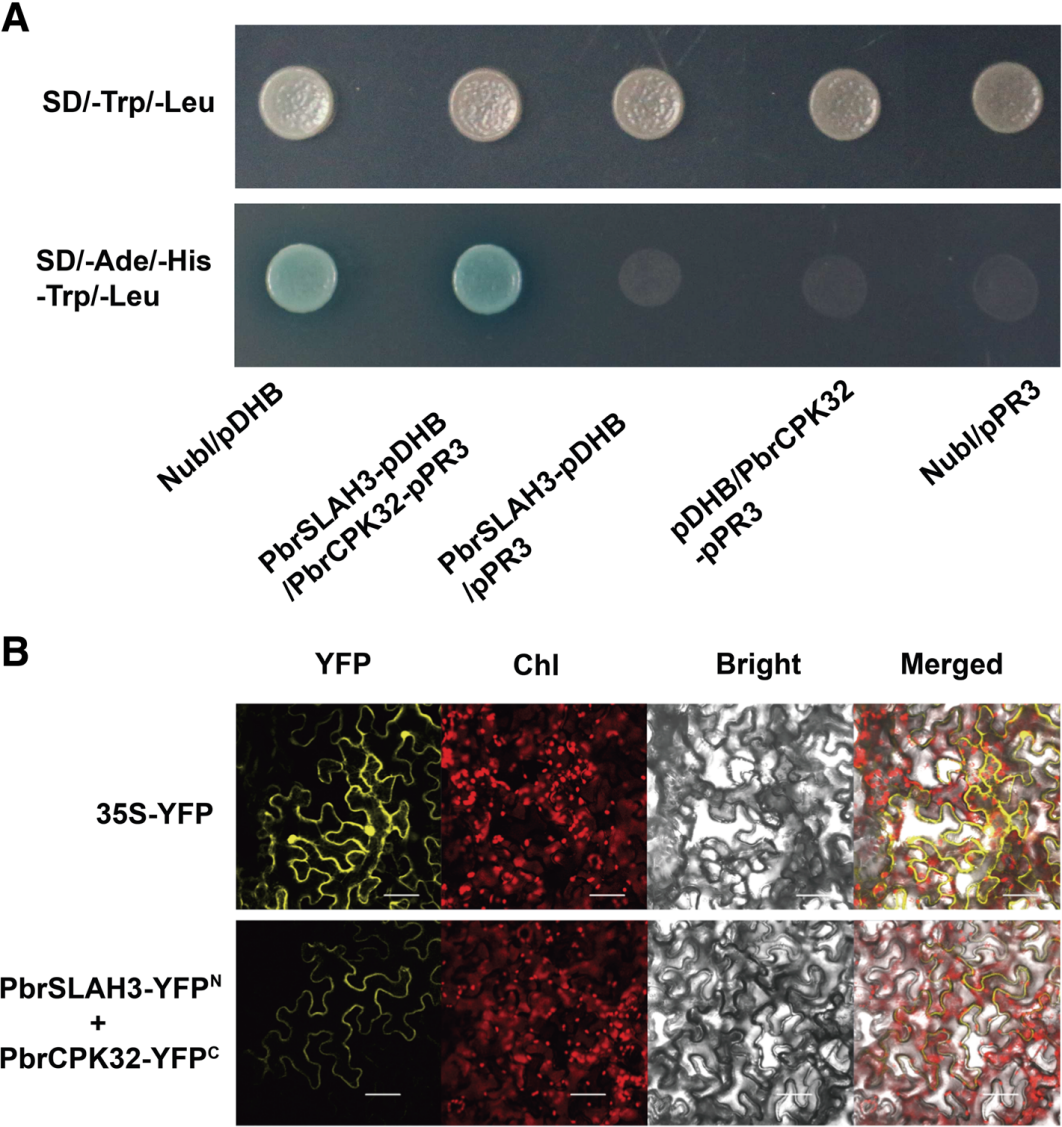
Interaction between PbrSLAH3 and PbrCPK32 as assessed by yeast two-hybrid and bimolecular fluorescence complementation assays. **a** Yeast strain NMY51 co-transformed with the *PbrSLAH3* bait vector and *PbrCPK32* prey vector, along with a positive control (co-transformed NubI vector and pDHB vector) were grown in medium lacking Ade, His, Trp and Leu. The negative control could not grow in the same medium. **b** YFP indicates yellow fluorescence protein; Chl: chlorophyll; Bright: bright-field image of *Nicotiana benthamiana* leaves infiltrated with *Agrobacterium tumefaciens*; Merge: digital merge of bright field and fluorescent images. Bar = 10 μm

Given PbrSLAH3 is actived by AtCPK21, we hypothesized that PbrSLAH3 may be regulated by orthologes of AtCPK21 in pear. We initially constructed a phylogenetic tree based on DNA sequences in pear to analyze associations between PbrSLAH3 and CPKs owing to the characteristic interactions between CPKs and anion channel proteins in *Arabidopsis* (Additional file 1: Figure S4 and Table S2). We identified 17 candidate PbrCPK proteins that may interact with PbrSLAH3 in pear. Then, a yeast two-hybrid experiment was firstly performed to assess the ability of PbrSLAH3 to interact with each of the PbrCPKs*.* The full-length of *PbrSLAH3* fused with the pDHB-C and the *PbrCPK*s fused with the pPR3-N vector were then co-transformed into the yeast strain NMY51 using the lithium acetate method, respectively. Co-transformed yeast cells were first selected on plates containing medium lacking Trp and Leu to confirm the introduction of the *PbrSLAH3*-pDHB-C and *PbrCPKs*-pPR3-N plasmids into the yeast cells (Additional file 1: Figure S5). The positive clones were then transferred to selective plates lacking Trp, Leu, His and Ade. As shown in Additional file 1: Figure S5, yeast cells co-transformed with the positive controls (NubG and pDHB-C) and interactive genes (*PbrSLAH3*-pDHB-C and *PbrCPK32*-pPR3-N) could grow on the selective medium (SD/−Trp-Leu-His-Ade). However, yeast cells harboring *PbrSLAH3*-pDHB-C and the other *PbrCPKs*-pPR3-N plasmids could not grow on the selective media. To further confirm the interaction between PbrSLAH3 and PbrCPK32, control experiments were performed to rule out self-activation. As shown in Fig. 4a, neither yeast cells harboring *PbrSLAH3*-pDHB-C and pPR3-N nor pDHB-C and *PbrCPK32*-pPR3-N could grow on the selective media. Only *PbrSLAH3*-pDHB-C and *PbrCPK32*-pPR3-N together could grow on the selective media (SD/−Trp-Leu-His-Ade; Fig. 4a). In addition, *PbrCPK32* is mainly expressed in the root system, and the subcellular localization study displayed that the PbrCPK32-GFP fusion protein is mostly distributed in the cell membrane (Additional file 1: Figure S6). In all, these results revealed that PbrSLAH3 could interact with PbrCPK32 in the plasma membrane.

To further confirm the interaction between PbrSLAH3 and PbrCPK32 in vivo, BiFC assays was performed (Fig. 4b). *PbrSLAH3* tagged with a split YFP^N^-terminal fragment (nYFP) and *PbrCPK32* tagged with an YFP^C^-terminal fragment (cYFP) were transiently co-infiltrated into epidermal cells of *N. benthamiana* leaves using *Agrobacterium*. As indicated in Fig. 4b, a strong YFP fluorescent signal was detected in the plasma membrane of epidermal cells when co-expression of *PbrSLAH3*-YFP^N^ and *PbrCPK32*-YFP^C^ or expression 35S-YFP alone (as a positive control), while no YFP fluorescent signal was observed in cells co-expressing of *PbrSLAH3*-YFP^N^ and YFP^C^ or *PbrCPK32*-YFP^C^ and YFP^N^ (Additional file 1: Figure S7). Thus, the BiFC assays further confirmed the interaction between PbrSLAH3 and PbrCPK32 in the plasma membrane.

### *PbrSLAH3* mediates S-type anion currents when co-expressed with *CPKs* in *Xenopus* oocytes

**Fig. 5:**
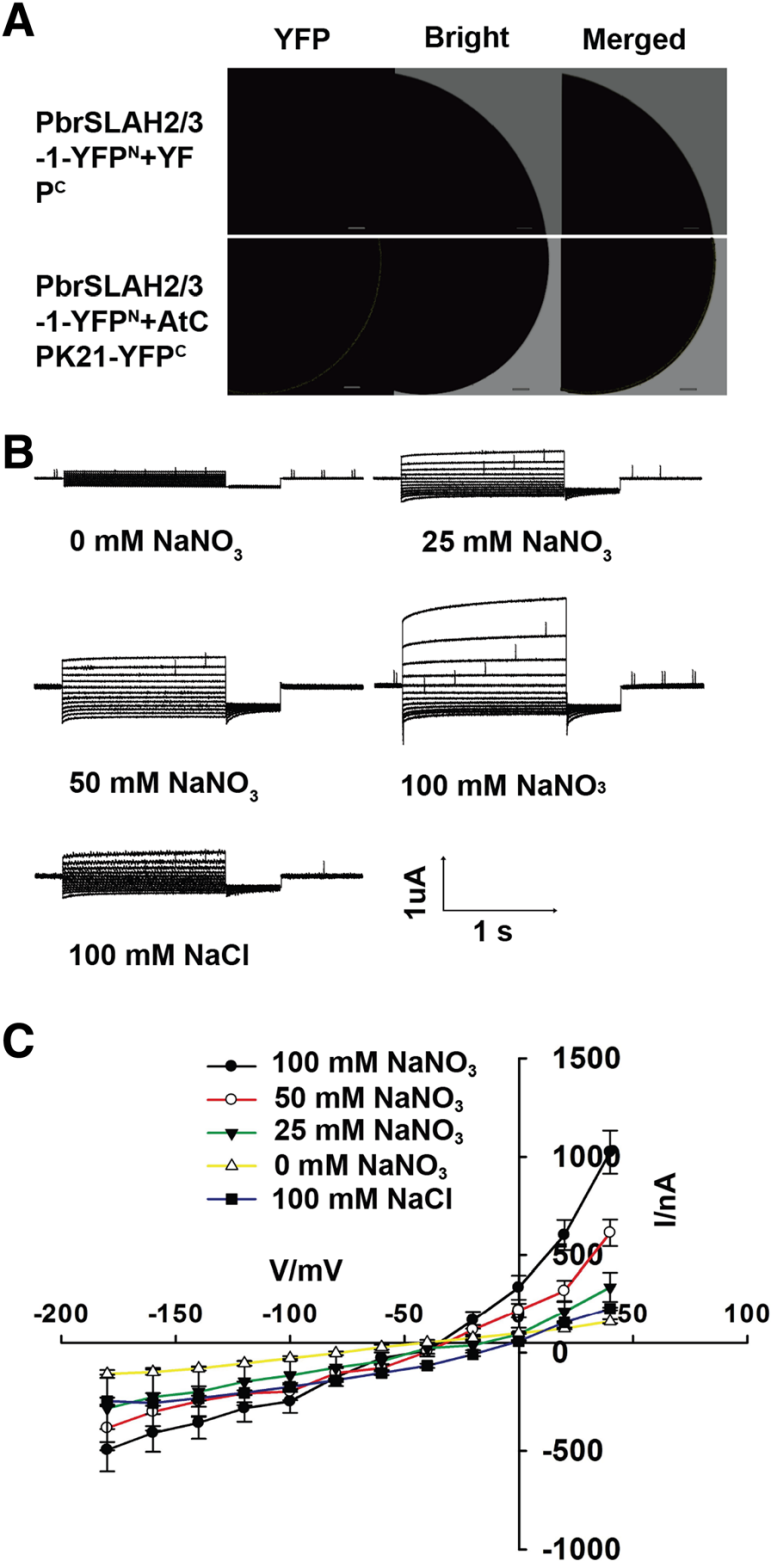
Electrical properties of *PbrSLAH3* co-expressed with *AtCPK21* in *Xenopus laevis* oocytes. **a** YFP fluorescence was emitted from *Xenopus* oocytes when *PbrSLAH3* was co-expressed with *AtCPK21*, but no YFP fluorescence was observed after the injection of *PbrSLAH3-YFP* alone. **b** Representive macroscopic anion currents were recorded in different concentrations of external NO_3_^−^ and Cl^−^ when *PbrSLAH3* was co-expressed with *AtCPK21*. Currents were recorded with 1.5-s voltage pulses ranging from − 180 mV to + 40 mV with 20-mV increments, followed by a 0.5-s voltage pulse to − 120 mV, the holding potential was 0 mV (*n* ≥ 5, means ± SE). **c** Steady state currents (Iss) plotted against the membrane voltages in the presence of different external NO_3_^−^ concentrations (n ≥ 5, means ± SE)

Since AtCPK21 interacts with PbrSLAH3, we further tested whether this protein kinase regulates the activity of PbrSLAH3. When this putative anion channel was expressed alone in *Xenopus* oocytes, no YFP fluorescence signal was detected and no anion currents could be measured (Fig. 5a and Additional file 1: Figure S8). However, when *PbrSLAH3* was co-expressed with *AtCPK21*, YFP fluorescence was emitted from oocytes and typical anion currents were recorded in the presence of NO_3_^−^ with membrane polarization (Fig. 5a).

In line with the functional characteristics of AtSLAH3, which is a preferential NO_3_^−^-permeable channel that is 20 times more permeable to NO_3_^−^ than Cl^−^, *PbrSLAH3* is also a preferential NO_3_^−^-permeable channel. The magnitudes of the anion currents were greater in the nitrate-bath medium than that under the same concentration of chlorine. In addition, the anion currents were largely dependent on the extracellular NO_3_^−^ concentrations (Fig. 5b). As shown in Fig. 5c, typical anion currents were elicited from oocytes in the presence of 25 mM nitrate medium. Although they were rather low, the anion currents increased 133 and 266% when the NO_3_^−^ concentration reached up to 50 mM and 100 mM, respectively. Together, in the presence of extracellular nitrate solution, PbrSLAH3 mediates S-type anion currents when co-expressed with AtCPK21 in *Xenopus* oocytes.

**Fig. 6:**
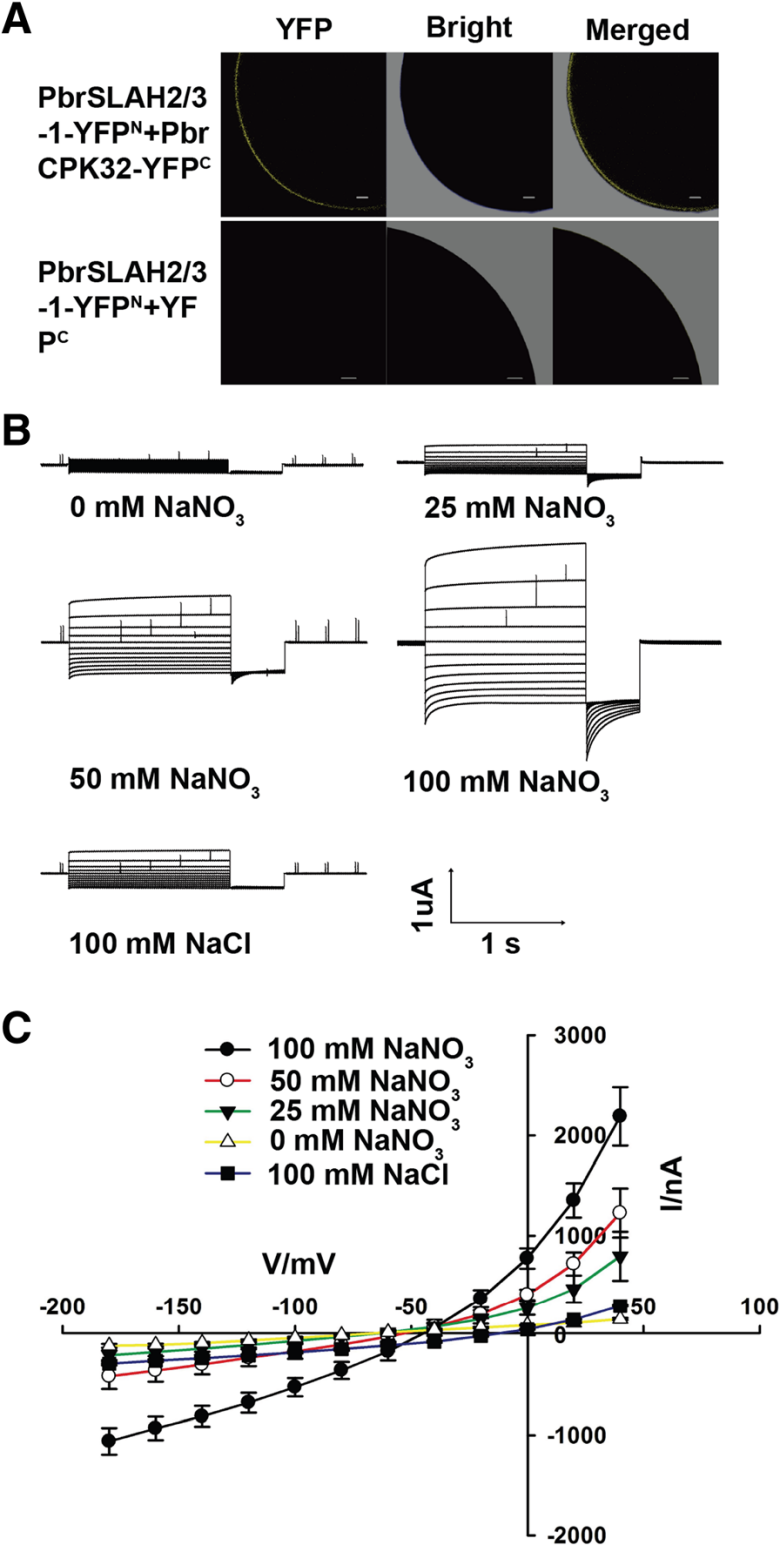
Electrical properties of *PbrSLAH3* co-expressed with *PbrCPK32* in *Xenopus* oocytes. **a** YFP fluorescence was observed in *Xenopus* oocytes when *PbrSLAH3* was co-expressed with *PbrCPK32*, while no YFP fluorescence was detected when *PbrSLAH3-YFP* cRNA alone was injected. **b** Representive macroscopic anion currents were recorded in different concentrations of external NO_3_^−^ and Cl^−^ when *PbrSLAH3* was co-expressed with *PbrCPK32*. Currents were recorded with 1.5-s voltage pulses ranging from − 180 mV to + 40 mV with 20-mV increments, followed by a 0.5-s voltage pulse to − 120 mV, the holding potential was 0 mV. (*n* ≥ 5, means ± SE). **c** Steady state currents (Iss) plotted against the membrane voltages in the presence of different external NO_3_^−^ concentrations (*n* ≥ 5, means ± SE)

Due to PbrSLAH3 was activated by AtCPK21 and an interaction was observed between PbrSLAH3 and PbrCPK32, we hypothesized that the activity of PbrSLAH3 was regulated by PbrCPKs in pear. To test this hypothesis, *PbrSLAH3* was injected alone or coinjected with *PbrCPK32* in the *Xenopus* oocytes. *PbrSLAH3* injected alone could not mediate the anion currents and no specific YFP fluorescence was emitted from *Xenopus* oocytes (Fig. 6a and Additional file 1: Figure S8). However, a YFP fluorescence signal was detected with a laser microscope (Fig. 6a) and macroscopic currents were elicited in the *Xenopus* oocytes when co-expression the *PbrSLAH3* with *PbrCPK32* (Fig. 6b). The I–V curves plotted against different concentrations of NO_3_^−^ showed that the magnitudes of the anion currents mediated by PbrSLAH3 were strongly dependent on the external NO_3_^−^ concentrations. However, only small macroscopic currents were induced by extracellular Cl^−^. Furthermore**,** the rate of NO_3_^−^ decrements was higher for 35S:*PbrSLAH3* plants than that of the *slah3–3* mutant *Arabidopsis*, based on NO_3_^−^ measurements from culture solution (Additional file 1: Figure S9). However, the rate of Cl^−^ decrements was barely changed between 35S:*PbrSLAH3* plants and *slah3–3* mutant *Arabidopsis,* based on Cl^−^ measurements from culture solution (Additional file 1: Figure S9), which indicated that overexpression of *PbrSLAH3* was greatly permeable NO_3_^−^ than Cl^−^ in the root. Taken together these results demonstrated that PbrSLAH3 was activated by PbrCPK32 and exhibited highly nitrate selectivity without obvious permeability to chloridion.

## Discussion

Nitrate is an important nutrient, and is also widely used as a signaling molecule during plant growth and development [14]. Following the up-take of nitrate by plasma membrane-localized nitrate transporters, nitrate is translocated into the vasculature for long-distance transport. It has been reported that *SLAC/SLAH* family genes could meditate the NO_3_^−^ efflux and may participate in NO_3_^−^ transport [20, 22, 25]. Considering *AtSLAC1* is exclusively expressed in guard cells, the other four *AtSLAH* (*SLAH1* to *SLAH4*) genes, which are predominantly expressed in root, are targets for NO_3_^−^ translocation in *Arabidopsis* root or from the root to shoot.

Based on previous reports about *SLAC/SLAH* genes expression patterns and functional characteristics [12, 28], we hypothesized that some members of the *SLAC/SLAH* family in pear may be involved in nitrate transport in root or from the root to the shoot, and may play an important role in determining the nitrogen use efficiency of the pear. In this study, four putative *SLAC/SLAH* genes were identified in the *Pyrus* genome. Surprisingly, it was found that orthologs of *AtSLAC/SLAH* in pear did not phylogenetically cluster with *AtSLAH1* and *AtSLAH4*. While, the expression patterns of *PbrSLAC/SLAH* are consistent with previous reports [25], *PbrSLAC1* is strongly expressed in leaves, and *PbrSLAH3* displays a higher expression level than other *SLAH* genes in pear root. Therefore, we presumed that PbrSLAH3 may predominantly participate in NO_3_^−^ transport in pear root. Here, the functional characteristics of *PbrSLAH3* were mainly studied using complementation lines and a *Xenopus* oocyte heterogeneous expression system.

The predominant expression of *PbrSLAH3* in root was further confirmed by RT-PCR and GUS staining assays, which demonstrated that it was also expressed in guard cells and pollen tubes, suggesting that *PbrSLAH3* may have diverse functions similar to those of *AtSLAH3*. Furthermore, phenotype assays showed that *PbrSLAH3* also rescues the ammonium toxicity phenomenon of the *slah3–3* mutant under low NO_3_^−^/high NH_4_^+^ conditions similar to that of the *AtSLAH3*. Taken together, we presumed that *PbrSLAH3* together with *AtSLAH3* and other orthologes from different species may be used as promising targets for crop enhancement, and they may serve as potential targets for transgenic breeding approaches to improve nitrogen use efficiency and reduce dependency on nitrogen fertilizers.

Post-translational modifications of transport proteins play key roles in the regulation of anion transport activities. For instance, AtSLAC1 and AtSLAH3 were activated by the Ca^2+^-independent protein kinase OST1 [29–31], as well as by AtCPKs [24, 29, 32]. SLAH2 was activated by CPK21 and CIPK23 kinases [22]. In this study, PbrSLAH3 was confirmed to interact with PbrCPK32 through yeast two-hybrid and BiFC assays. Furthermore, the electrophysiological experiments demonstrated that PbrSLAH3’s activation requires phosphorylation. When *PbrSLAH3* alone injection into *Xenopus* oocytes, no currents were elicited, but when *PbrSLAH3* and *AtCPK21* were co-expressed in the *Xenopus* oocytes, typical anion currents were recorded in the presence of NO_3_^−^. However, not all isolated anion channels from different species dependent on the phosphorylation process. For example, PttSLAH3 isolated from poplar is activated independent of phosphorylation by protein kinases [10]. In addition, the silent *SLAH1* subunit gates *SLAH3* open even in the absence of calcium-dependent kinases when co-expression the *SLAH1* and *SLAH3* in *Xenopus* oocytes [20]. In line with the function characteristic of the AtSLAH3, PbrSLAH3 was strongly dependent on the external concentration of NO_3_^−^ and showed a high selectivity for nitrate over chloride. However, the biological functions of PbrSLAH3 and PbrCPK32 in pear are still unknown.

## Conclusions

S-type anion channels play an important role in nitrate translocation, revealing another manner to expand the transport of nitrate anions. However, the function characteristics of *PbrSLAC/SLAH* in pear have not been clarified. In this study, four members of S-type anion channels were identified in pear using *Arabidopsis SLAC/SLAH* sequences as queries. In combination with homologous similarity and expression analyses, *PbrSLAH3* was selected as a candidate gene involved in NO_3_^−^ transport in pear root. The physiological function of *PbrSLAH3* was investigated by complementation lines, growth assays suggested that *PbrSLAH3* participates in the alleviation of ammonium toxicity. PbrSLAH3 interaction with PbrCPK32 was firstly confirmed by yeast two-hybrid and BiFC assays, and PbrSLAH3 activity was controlled through phosphorylation by CPKs was further verified by co-expression it with *AtCPK21* or *PbrCPK32* in *Xenopus* oocytes. Furthermore, electrophysiological and physiology experiment results showed that PbrSLAH3 was more permeable to NO_3_^−^ than Cl^−^. Taken together these results, we concluded that PbrSLAH3 crossing-talk with PbrCPK32 may participate in transporting NO_3_^−^ in pear root.

## Methods

### Plant materials and growth conditions

The Columbia (Col-0) line of *Arabidopsis* was used as the wild-type (Col-0). The *slah3–3* (SALK_106054) mutant seed was ordered from the *Arabidopsis* Biological Resource Center. Homozygous mutant plants were isolated by PCR with corresponding primers, as listed in Additional file 1: Table S1. For seed harvest, *Arabidopsis* plants were grown in a potting soil mixture (rich soil:vermiculite = 2:1, v/v) and kept in growth chambers with a light/dark regime of 14/10 h, at 28/22 °C with 75% relative humidity and a light intensity of 800 μmol m^− 2^ s^− 1^. For nitrate treatment experiments, surface-sterilized seeds were germinated and grown on 1/2 N-free Murashige and Skoog medium (pH 5.8) containing different concentrations (0–20 mM) of KNO_3_ with or without 20 mM ammonium chloride. Plates were kept at 4 °C for 3 d before seed germination. All plants were grown vertically under axenic conditions in growth chambers as described above. Plants were photographed with a Canon EOS 80D camera (Canon, Tokyo, Japan). Statistical analyses of the data were performed using the SPSS statistical package (SPSS Version 17.0 for Windows; SPSS Inc., Chicago, IL).

### Bioinformatics analysis of *PbrSLAC/PbrSLAH*

The protein sequences of AtSLAC1 (AT1G12480), AtSLAH1 (AT1G62280), AtSLAH2 (AT4G27970), AtSLAH3 (AT5G24030) and AtSLAH4 (AT1G62262) were used as queries to perform a BLAST algorithm-based search of the pear genome database (http://peargenome.njau.edu.cn/). Based on our previous research, four PbrSLAC/SLAH protein sequences were identified [33]. They were aligned with orthologs in *Arabidopsis*, and a phylogenetic tree was generated by MEGA (version 6.0) software with the neighbor-joining method and a bootstrap test of 1000 replicates [34].

### RNA isolation and qRT-PCR assays

Total RNA was extracted from different parts (root, stem, leaf, flower, fruit, pollen grain and pollen tube) of pear using an RNA extracting kit (RNAsimply Total RNA Kit, Tiangen, Beijing, China) according to the manufacturer’s protocol. Then approximately 2 μg total RNA was used for first-strand cDNA synthesis using TransScript®One-Step gDNA Removal and cDNA Synthesis SuperMix (TRANSGEN, Beijing, China). Three biological and three technical replicates were processed for the qRT-PCR assays, which was performed in 20 μL reaction mixture containing 80–100 ng cDNA, 200 nM of each primer (Additional file 1: Table S1) and 10 μL LightCycler 480 SYBRGREEN I Master Mix (Roche, Basel, Switzerland). All reactions were carried out in a CFX96 Real-Time System (Roche), following a three step standard protocol (45 cycles of 10 s at 95 °C, 30 s at 60 °C and 30 s at 72 °C), followed by a melt curve analysis. The expression levels of *PbrSLAC/SLAH* genes were calibrated by the 2^–ΔΔCt^ method using *PbrUBQ* and *PbrTUB* as reference genes [35]. RT-PCR was carried out on a Veriti™ 96-Well Thermal cycler (Bio-Rad, Richmond, CA, USA) using Taq DNA Polymerase (New England Biolabs, Beverly, MA, USA). *PbrUBQ* was used as the reference gene. PCR products were separated on a 2% (w/v) agarose gel stained with ethidium bromide.

### Subcellular localization assays

To test the subcellular localization of PbrSLAH3 and its regulatory protein, the full-length coding sequences of *PbrSLAH3* and *PbrCPK32* were amplified with the corresponding primers, respectively, which are listed in Additional file 1: Table S1. The purified PCR products were independently inserted into the pCAMBIA1302 vector under the control of the CaMV 35S promoter and the vector is detailed in xie et al [36]. The recombinant *plasmids* 35S:*PbrSLAH3*-green fluorescence protein (GFP) and 35S:*PbrCPK32*-GFP, as well as the control *plasmid* 35S:GFP, were independently introduced into *Nicotiana benthamiana* leaves by *Agrobacterium*-mediated transformation. Transformed leaves were stained with double-distilled water supplemented with 5 μM FM4–64 (Invitrogen) for 15 min, and the GFP signal was detected with a Zeiss LSM 780 Image Browser (Carl Zeiss Inc.) 3 d after transformation.

### BiFC assays

The full-length cDNAs of *PbrSLAH3* and *PbrCPK32* were each inserted independently into the pYFP-N and pYFP-C vectors [37] and sequenced. Then, *N. benthamiana* leaves were transiently transformed using the *Agrobacterium* infection method with different combinations of these vectors. After 3 d, the YFP expression signals emitted from *N. benthamiana* leaves were observed with a Zeiss LSM 780 Image Browser (Carl Zeiss Inc.).

### Yeast two-hybrid assays

To screen for interactions between PbrSLAH3 and PbrCPKs, the coding sequence of *PbrSLAH3* was cloned into the pDHB-C vector, and the full-length *PbrCPK* cDNAs were independently cloned into the pPR3-N vector [38]. Next, *PbrSLAH3*-pDHB-C and *PbrCPK*-pPR3-N plasmids were co-transformed into the yeast strain NMY51 using the lithium acetate method. NubG co-transformed with pDHB-C was used as a positive control, and NubG co-transformed with pPR3-N was used as a negative control. Then, co-transformed yeast cells were grown on selective medium lacking leucine and tryptophan (SD/−Trp-Leu) and were cultured at 28 °C. Colonies from SD/−Trp-Leu plates were transferred to plates lacking leucine, tryptophan, histidine and adenine (SD/−Trp-Leu-His-Ade) and dyed with X-gal to test for possible interactions.

### Vector construction and *Arabidopsis* transformation

The *PbrSLAH3* promoter fragment (~ 2 kb) was cloned into the pBasta-GUSGW vector [39], and *PbrSLAH3* was cloned into the pCAMBIA1302 vector [36]. Then, *PbrSLAH3*::GUS and *PbrSLAH3*-pCAMBIA1302 recombination vectors were transformed into Col-0 *Arabidopsis* plants and *slah3* mutant plants, respectively. The *Arabidopsis* transformation was performed using the floral dip method with *Agrobacterium* GV3101 [40]. The seeds of the transgenic plants were collected and then plated on half-strength MS medium containing 30 mg/L of hygromycin. Positive seedlings were verified by PCR with primers listed in Additional file 1: Table S1, and T3 homozygous transgenic lines were used for the later GUS assays and phenotypic analysis.

### GUS assays

GUS staining was carried out according to the method described by Jefferson et al [40, 41]. Two-week-old seedlings, flowers and siliques were vacuum-infiltrated for 30 min in the staining solution (GUS staining kit, Pulangsai, Beijing, China) followed by incubation at 37 °C for 3 h. After gradient decolorization by alcohol, the samples were photographed under a microscope with a Canon EOS 80D camera (Canon, Tokyo, Japan).

### Double-electrode voltage-clamp studies (oocyte recordings)

*PbrSLAH3* cDNA fused with pYFP-C and *PbrCPK32* cDNA fused with pYFP-N were independently cloned into the pT7TS vector using homologous recombination technology [42]. The capped and polyadenylated cRNA of these two fusion genes were synthesized in vitro using a MESSAGE kit (Ambion, Austin, Texas, USA). Then, 50 nL cRNA mixture of the target genes (*PbrSLAH3*-YFP-C and *PbrCPK32*-YFP-N) or 50 nL of deionized water (control) was injected into matured oocytes, which were then cultured in ‘ND96’ solution (96 mM NaCl, 2 mM KCl, 1.8 mM CaCl_2_, 1 mM MgCl_2_ and 5 mM HEPES, adjusted to pH 7.4 with NaOH) until electrophysiological recordings were taken. Then, 3–5 d after cRNA injections, whole-oocyte currents were recorded using the two-electrode voltage-clamp technique. The voltage-clamp amplifier was an Axoclamp 900A (Axon Instruments, Foster City, CA, USA). Electrodes were filled with 3 M KCl, and the standard solution contained 5 mM Tris/MES (pH 7.5), 1 mM Ca(gluconate)_2_, 1 mM Mg(gluconate)_2_, 100 mM NaNO_3_ and 1 mM LaCl_3_. To balance the ionic strength, nitrate or chloride variations were compensated with sorbitol. Osmolality was adjusted to 220 mos- mol/kg with D-sorbitol. The voltage-clamp protocols are described in the figure legends, and data acquisition and data analyses were performed using pClampfit 10.3 (Molecular Devices) and Sigmaplot 12.5 software (Jandel Scientific, Erkrath, Germany), respectively. Absorption or transport.

### Determination of the NO_3_^−^ and cl^−^ contents

The 35S:*PbrSLAH3* and *slah3–3* mutant *Arabidopsis* seeds were plated on solid 1/2 MS medium. Two-week-old seedlings were pre-cultured in the 72 holes rectangular box containing 500 mL of liquid 1/2 MS medium for 12 d. In the process of pre-culture, the nutrient solution was replaced with fresh solution every 4 d. After the pre-culture, seedlings were treated with the 1/2 NO_3_^−^ −free and Cl^−^-free MS for 3 days, and then were transferred into modified liquid 1/2 MS containing 5 mM NO_3_^−^ and 5 mM Cl^−^. 180 seedlings were used per treatment, with three replications. Concentrations of NO_3_^−^ and Cl^−^ were determined in the liquid 1/2 MS solution at 0 h, 6 h, 12 h, 24 h, 48 h and 96 h after the transferring, respectively, which are based on the method described by Cataldo et al [43] and Gilliam et al [44]. In addition, the volume of nutrient solution needs to be replenished to 500 mL with double distilled water before each sampling. Statistical analyses of the data were performed using the SPSS statistical package (SPSS Version 17.0 for Windows; SPSS Inc., Chicago, IL). Three biological replicates and three technical replicates were processed, and all plants were grown in chambers with a light/dark regime of 14/10 h, at 28/22 °C with 75% relative humidity and a light intensity of 800 μmol m^− 2^ s^− 1^.

## Additional file

Additional file 1: **Figure S1.** Changes in PbrSLAH3 expression in response to re-supplying nitrate in pear root after nitrate starvation. **Figure S2.** PbrSLAH3 rescues the ammonium toxicity of *slah3–3* mutant plants under high-ammonium/low-nitrate conditions. **Figure S3.** PbrSLAH3 interaction with AtCPK21 was confirmed by bimolecular fluorescence complementation assays. **Figure S4.** Phylogenetic tree of calcium-dependent proteins in pear and in *Arabidopsis*. **Figure S5.** The interaction between *PbrSLAH3* and *PbrCPKs* was assessed by the yeast two-hybrid assays. **Figure S6.** The subcellular localization of *PbrCPK32* in plasma membrane and the expression profiles of the PbrCPK32 genes in different parts of the pear were analyzed by qRT-PCR. **Figure S7.** No fluorescence was detected when *PbrSLAH3* or *PbrCPK32* was expressed alone in *N. benthamiana* leaves (negative control). **Figure S8.** Representive macroscopic anion currents were recorded in 50 mM NaNO_3_ solution after independently injects of H_2_O and PbrSLAH_3_ alone. **Figure S9.** Comparative analysis of NO_3_ and Cl transport between *35S:PbrSLAH3* and *slah3–3* mutant plants. **Table S1.** Primers used in this study. **Table S2.** CPK family genes identified in pear. (PDF 1355 kb)

## Abbreviations

ABA: Abscisic acid
Ade: Adenine
BiFC: Bimolecular fluorescence complementation
CPK: Calcium-dependent protein kinase
GFP: Green Fluorescent Protein
GUS: *β*-Glucuronidase
His: Leucine
Leu: Leucine
OST1: Stomatal opening factor 1
qRT-PCR: Quantitative real-time polymerase chain reaction
RT-PCR: Real-time polymerase chain reaction
S-type anion channels: S-type anion channels
Trp: Tryptophan
YFP: Yellow Fluorescent Protein
