# Supplementary material for: PbrSLAH3 is a nitrate-selective anion channel which is modulated by calcium-dependent protein kinase 32 in pear
Source: BMC Plant Biol. 2019 May 8;19:190. doi: 10.1186/s12870-019-1813-z (PMC6507222; doi:10.1186/s12870-019-1813-z)
Supplement: Supplementary file 1 — Figure S1. Changes in PbrSLAH3 expression in response to re-supplying nitrate in pear root after nitrate starvation. Figure S2. PbrSLAH3 rescues the ammonium toxicity of slah3–3 mutant plants under high-ammonium/low-nitrate conditions. Figure S3. PbrSLAH3 interaction with AtCPK21 was confirmed by bimolecular fluorescence complementation assays. Figure S4. Phylogenetic tree of calcium-dependent proteins in pear and in Arabidopsis. Figure S5. The interaction between PbrSLAH3 and PbrCPKs was assessed by the yeast two-hybrid assays. Figure S6. The subcellular localization of PbrCPK32 in plasma membrane and the expression profiles of the PbrCPK32 genes in different parts of the pear were analyzed by qRT-PCR. Figure S7. No fluorescence was detected when PbrSLAH3 or PbrCPK32 was expressed alone in N. benthamiana leaves (negative control). Figure S8. Representive macroscopic anion currents were recorded in 50 mM NaNO3 solution after independently injects of H2O and PbrSLAH3 alone. Figure S9. Comparative analysis of NO3 and Cl transport between 35S:PbrSLAH3 and slah3–3 mutant plants. Table S1. Primers used in this study. Table S2. CPK family genes identified in pear. (PDF 1355 kb) [file 12870_2019_1813_MOESM1_ESM.pdf]

PbrSLAH3 is a nitrate-selective anion channel which is modulated by calcium-dependent protein kinase 32 in pear

Running head: *PbrSLAH3* functions in nitrate transport.

Guodong Chen[#], Li Wang[#], Qian Chen, Kaijie Qi, Hao Yin, Peng Cao, Chao Tang, Xiao Wu, Shaoling Zhang, Peng Wang[*], Juyou Wu[*]

Center of Pear Engineering Technology Research, State Key Laboratory of Crop Genetics and Germplasm Enhancement, College of Horticulture, Nanjing Agricultural University, Nanjing 210095, China

[#]These authors contributed equally to this work

*Address correspondence to: Juyou Wu and Peng Wang

No 6. Tongwei Road, Nanjing, China.

Tel. 0086-25-84396580;

Fax.0086-25-84396485.

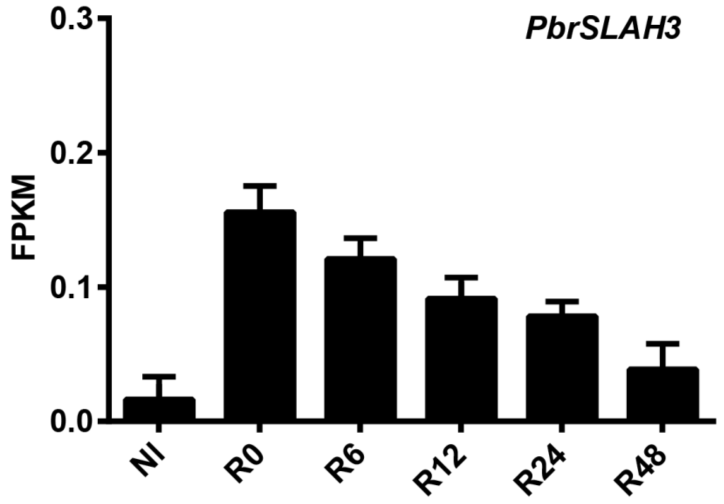

Figure S1 Changes in *PbrSLAH3* expression in response to re-supplying nitrate in pear root after nitrate starvation.

FPKM data presented are means ± SE from three independent biological experiments. Abbreviations used in this figure are as follows: Nl, pear root cultured in the normal solution; R0, R6, R12, R24 and R48 represents pear root resupplied with nitrate for 0, 6, 12, 24 and 48 h, respectively in normal nutrient solution after culturing under nitrate starvation (N-free) conditions for 5 d. FPKM, reads per kilobase of exon model per million mapped reads.

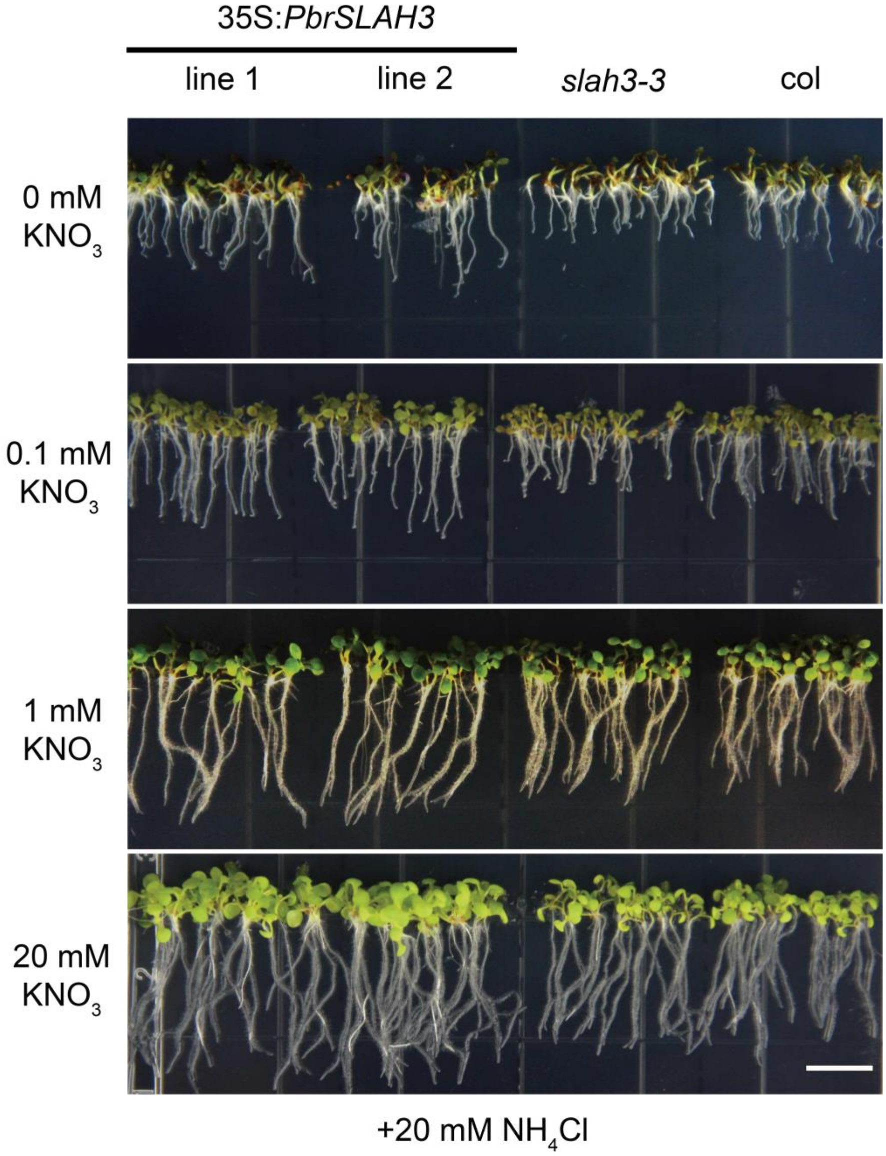

Figure S2 PbrSLAH3 rescues the ammonium toxicity of slah3-3 mutant plants under high-ammonium/low-nitrate conditions.

Growth phenotype testing among 7-d-old transgenic lines 1 and 2, wild-type (Col-0) and slah3-3 mutant seedlings was carried out. The seeds were geminated and grown on 1/2 N-free Murashige and Skoog (MS) medium supplemented with different concentrations of KNO_3 (0–20 mM) and 20 mM NH_4Cl. Bar = 0.5 cm.

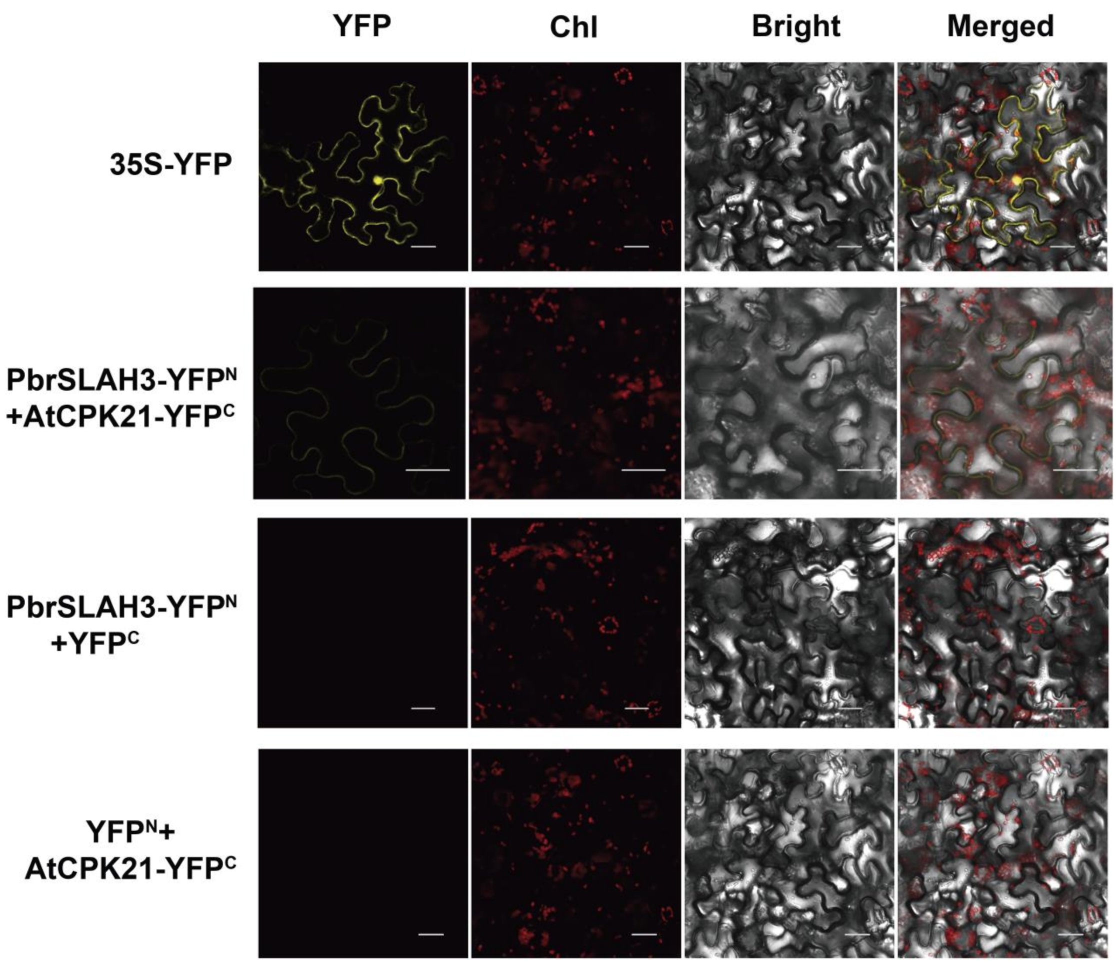

Figure S3 PbrSLAH3 interaction with AtCPK21 was confirmed by bimolecular fluorescence complementation assays.

YFP indicates yellow fluorescent protein; Chl: chlorophyll; Bright: bright-field image of *Nicotiana benthamiana* leaves infiltrated with *Agrobacterium tumefaciens*; Merge: digital merge of bright field and fluorescent images. Bar = 10 μm.

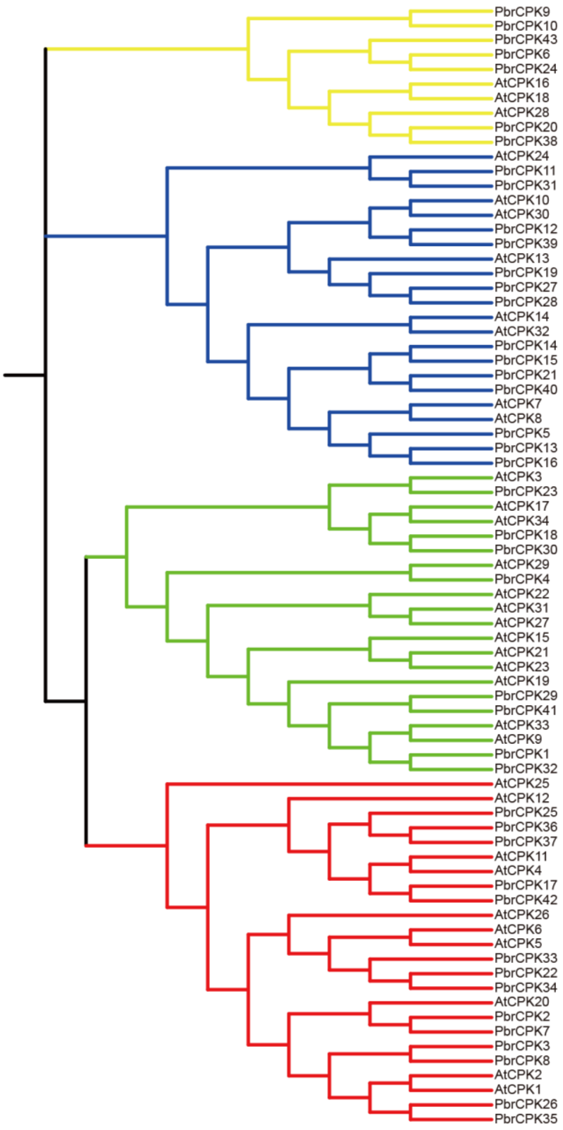

Figure S4 Phylogenetic tree of calcium-dependent proteins in pear and in *Arabidopsis*.

The protein sequences of AtCPKs were used as queries to perform a BLAST algorithm-based search of the pear genome database (http://202.195.250.6/wp/). Forty-three PbrCPKs protein sequences were identified. They were aligned with orthologs in *Arabidopsis*, and a phylogenetic tree was generated by MEGA6.0 (http://www.megasoftware.net/) with the neighbor-joining method and a bootstrap test of 1,000 replicates

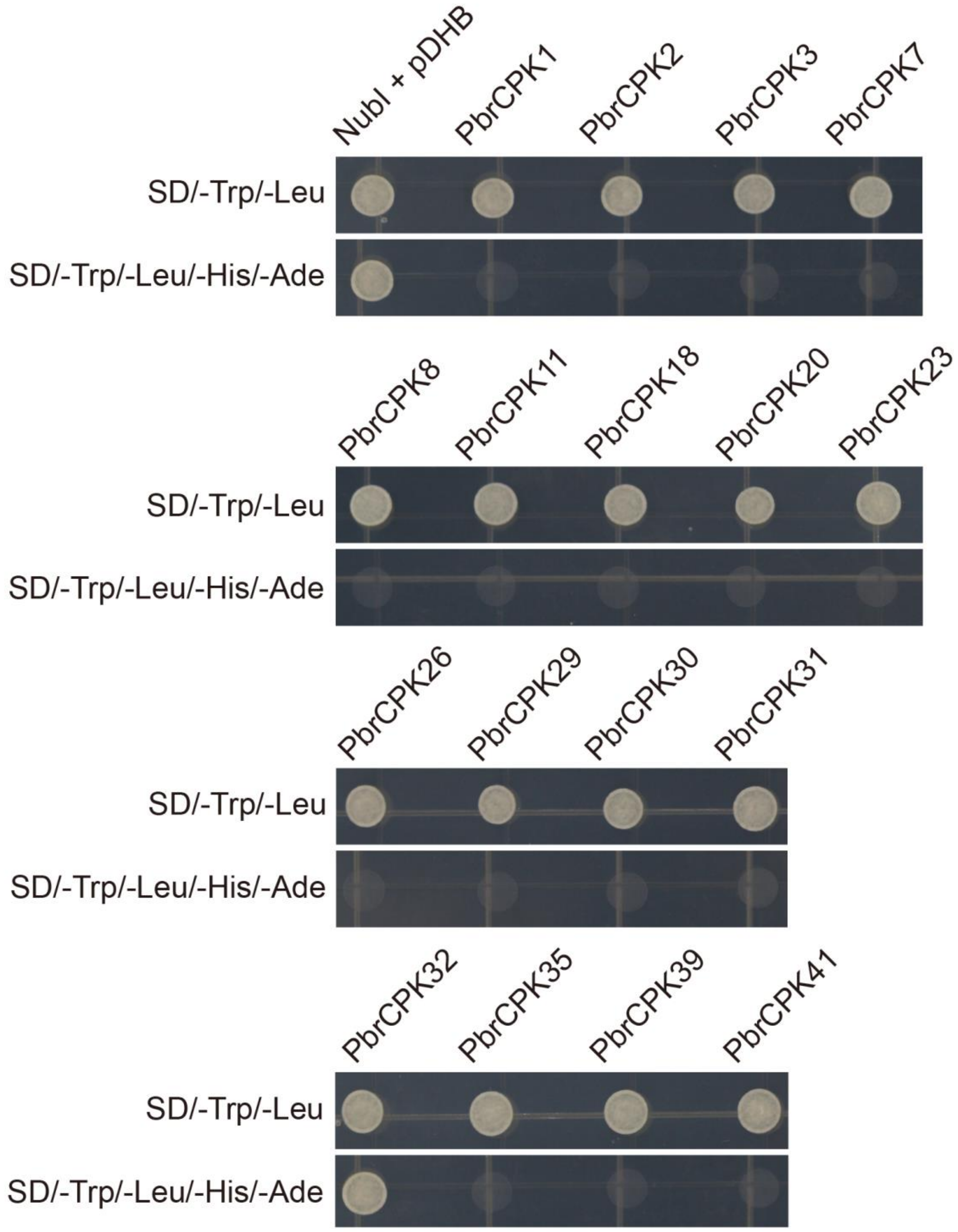

Figure S5 The interaction between *PbrSLAH3* and *PbrCPKs* was assessed by the yeast two-hybrid assays.

Yeast strain NMY51 cells were co-transformed *PbrSLAH3* bait vector and each of the *PbrCPKs* prey vector and grown in SD/−Leu−Trp plates. Then, positive clones were transferred to the SD/−Ade−His−Leu−Trp selective medium (below).

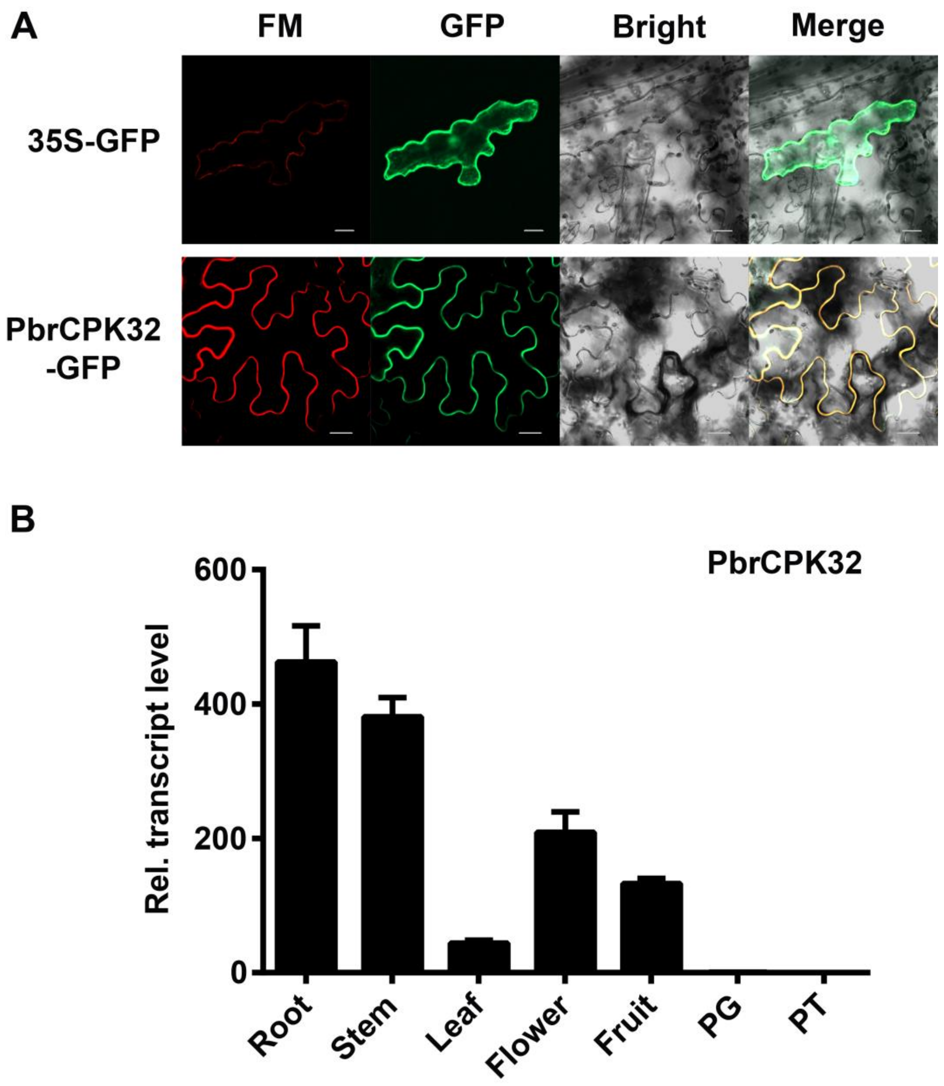

Figure S6 The subcellular localization of *PbrCPK32* in plasma membrane and the expression profiles of the *PbrCPK32* genes in different parts of the pear were analyzed by qRT-PCR.

A. GFP signaling emitted from the leaves of tobacco infected by *Agrobacterium* harboring *PbrCPK32*-GFP were detected with confocal microscopy. GFP: Green fluorescence of PbrSLAH3-GFP and PbrCPK32-GFP proteins; FM: Plasma membranes are stained red by FM4-64; Bright: The bright-field image of *Agrobacterium* tumefaciens-infiltrated tobacco leaves; Merge: The merged fluorescent images Bar=10μm. B. Total RNA was extracted from roots, stems, leaves, flowers, fruit, pollen grains and pollen tubes cultured for 5 h. The relative expression levels were obtained by reference to mRNA content of *PbrUBQ*. The data are shown as mean values ± SDs.

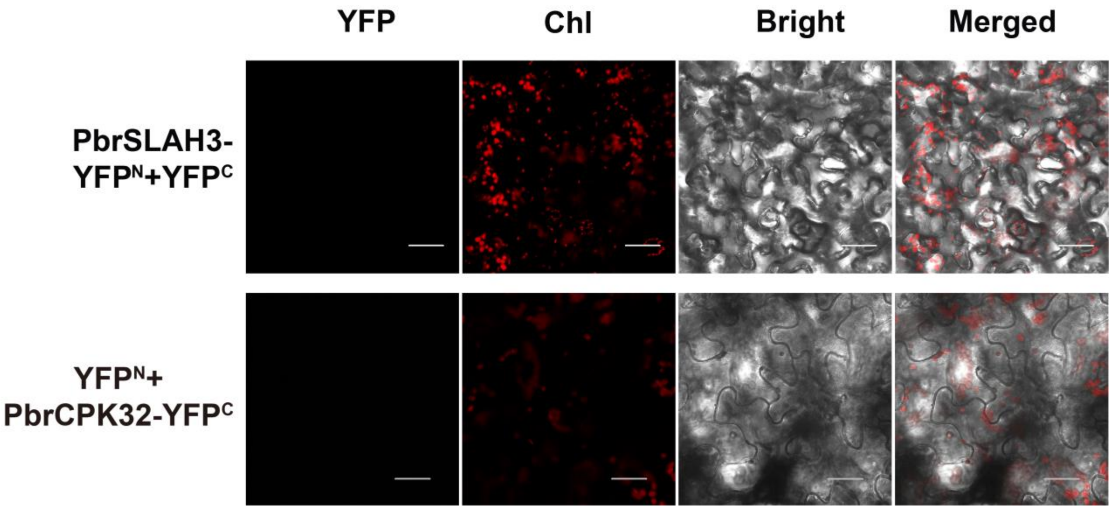

Figure S7 No fluorescence was detected when PbrSLAH3 or PbrCPK32 was expressed alone in *N. benthamiana* leaves (negative control).

No signaling was detected when expression the PbrSLAH3 or PbrCPK32 alone in *N. benthamiana* leaf (negative control). YFP indicates fluorescence of YFP; Chl: Red fluorescence of chlorophyll; Bright: the bright-field image of *Nicotianabenthamiana* leaves infiltrated with *Agrobacterium* tumefaciens; Merge is digital merge of bright field and fluorescent images. Bar=10μm.

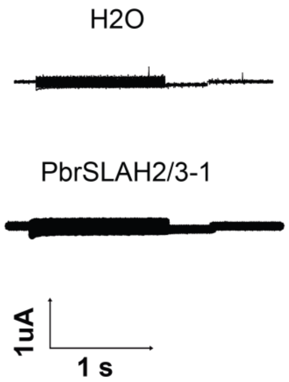

Figure S8 Representive macroscopic anion currents were recorded in 50 mM $NaNO_3$ solution after independently injects of H2O and PbrSLAH3 alone.

The currents were recorded with 1.5-s voltage pulses ranging from −180 mV to +40 mV, with 20-mV increments, followed by a 0.5-s voltage pulse to −120 mV. The holding potential was 0 mV.

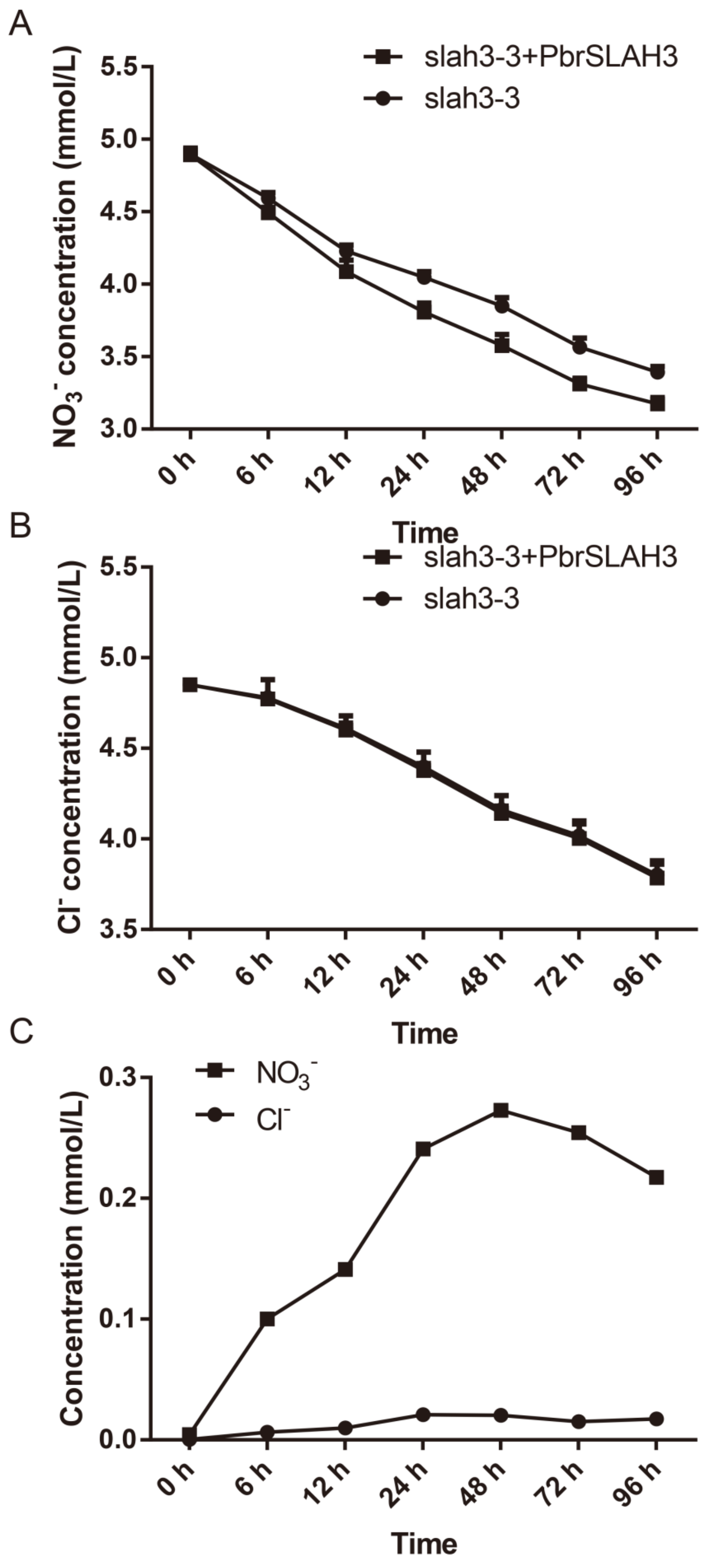
A
slah3-3+PbrSLAH3
slah3-3
NO3- concentration (mmol/L)
5.5
5.0
4.5
4.0
3.5
3.0
0 h
6 h
12 h
24 h
48 h
72 h
96 h
Time
B
slah3-3+PbrSLAH3
slah3-3
Cl- concentration (mmol/L)
5.5
5.0
4.5
4.0
3.5
0 h
6 h
12 h
24 h
48 h
72 h
96 h
Time
C
NO3-
Cl-
Concentration (mmol/L)
0.3
0.2
0.1
0.0
0 h
6 h
12 h
24 h
48 h
72 h
96 h
Time

Figure S9 Comparative analysis of NO_3^- and Cl^- transport between *35S*:*PbrSLAH3* and *slah3-3* mutant plants.

A. The NO_3^- decrements of the culture solution were measured at the time of 0, 6, 12, 24, 48, 72 and 96 hours respectively, at which the *35S:PbrSLAH3* and *slah3-3* mutant plants had been incubating. B. The Cl^- decrements of the culture solution were measured at the time of 0, 6, 12, 24, 48, 72 and 96 hours respectively, at which the *35S:PbrSLAH3* and *slah3-3* mutant plants had been incubating. C. The transport amounts of NO_3^- and Cl^- mediated by PbrSLAH3 was calculated by comparing the concentration changes of the two anions from the culture solution in which the 35S:*PbrSLAH3* and *slah3-3* mutant *Arabidopsis* were incubated. The data are presented as the means ± SDs of three replicates, and each replicate is based on measurements taken from three samples.

Table S1 Primers used in this study

Primers used to verify the T-DNA insertion lines and slah3-3		
LB1.3 (T-DNA primer for SALK lines)	BP	ATTTTGCCGATTTCGGAAC
slah3-3 (SALK_106054)	RP	TTTGTTTTCCTTCGCATATGC
slah3-3 (SALK_106054)	LP	ATCTCTTCTTGAGGCTGCGAC
Primers for qRT-PCR and semi-quantitative RT-PCR		
PbrUBQ-qRT-PCR	Forward	AAGCACAAGCACAAGAAGGT
PbrUBQ-qRT-PCR	Reversed	CTCTAATCAGCCAGCCTTC
PbrTUB-qRT-PCR	Forward	TGGGCTTTGCTCCTCTTAC
PbrTUB-qRT-PCR	Reversed	CCTTCGTGCTCATCTTACC
PbrSLAC1-qRT-PCR	Forward	GTTTCCGTAACTCACATTTCATTG
PbrSLAC1-qRT-PCR	Reversed	CCTTCCATGCCAACCCTAGT
PbrSLAH3-qRT-PCR	Forward	GCATTTCCCTTCTCACCCGA
PbrSLAH3-qRT-PCR	Reversed	CCAACTCTGGCCCTTCCAAT
PbrSLAH2/3-2-qRT-PCR	Forward	CAGTCCAGCTGAAGGAATTGAGA
PbrSLAH2/3-2-qRT-PCR	Reversed	CTTTTTGGCATTGGCTGGGA
PbrSLAH2/3-3-qRT-PCR	Forward	CTTTCCCGATGACTGGTGCT
PbrSLAH2/3-3-qRT-PCR	Reversed	ATTTGTCAACGTGTCCGAGT
PbrCPK32-qRT-PCR	Forward	CAGGGCACAGGCTGCACA
PbrCPK32-qRT-PCR	Reversed	TGGCCGGTGGAGTTCTCAGT
PbrUBQ-RT-PCR	Forward	AAGCACAAGCACAAGAAGGT
PbrUBQ-RT-PCR	Reversed	CTCTAATCAGCCAGCCTTC
PbrSLAC1-RT-PCR	Forward	GTTTCCGTAACTCACATTTCATTG
PbrSLAC1-RT-PCR	Reversed	CCTTCCATGCCAACCCTAGT
PbrSLAH3-RT-PCR	Forward	GCATTTCCCTTCTCACCCGA
PbrSLAH3-RT-PCR	Reversed	CCAACTCTGGCCCTTCCAAT
PbrSLAH2/3-2-RT-PCR	Forward	CAGTCCAGCTGAAGGAATTGAGA
PbrSLAH2/3-2-RT-PCR	Reversed	CTTTTTGGCATTGGCTGGGA
PbrSLAH2/3-3-RT-PCR	Forward	CTTTCCCGATGACTGGTGCT
PbrSLAH2/3-3-RT-PCR	Reversed	ATTTGTCAACGTGTCCGAGT
Primers used for GUS assays		
PbrSLAH3-GUS	HindIII-Forward	AAGCTTAAACATGACCAACAAAAGGAC
PbrSLAH3-GUS	BamH I-Reversed	GGATCCATTTTTGTTACTTTTCAGCATAAAAATT
Primers used for BiFC assays		
PbrSLAH3-nYFP	Xba I-Forward	TCTAGAATGAATTCTGGAAAACAGAAATCCC
PbrSLAH3-nYFP	BamH I-Reversed	GGATCCCACCATGGCATCTCGATGCAA
PbrCPK32-cYFP	Xba I-Forward	TCTAGAATGGGTTGCTGTAGCAGCAAAC
PbrCPK32-cYFP	BamH I-Reversed	GGATCCAAAGAGCATTGCTGGTTGTTGAGT

AtCPK21-cYFP	Xba I-Forward	TCTAGAATGGGTTGCTTCAGCAGTAAACA
AtCPK21-cYFP	BamH I-Reversed	GGATCCATGGAATGGAAGCAGTTTCCC
Primers used for Y2H assays		
PbrSLAH3-pDHB-C	SfiI-Forward	GGCCATTACGGCCATGAATTCTGGAAAACAGAAATCCC
PbrSLAH3-pDHB-C	SfiI-Reversed	GGCCGAGGCGGCCCACCATGGCATCTCGATGCAA
PbrCPK1-pPR3-N	SfiI-Forward	GGCCATTACGGCCATGGGTTGCCACAGCAGCAAAGA
PbrCPK1-pPR3-N	SfiI-Reversed	GGCCGAGGCGGCCAAAGAGCTTTGCTGGTTGTTGAG
PbrCPK2-pPR3-N	SfiI-Forward	GGCCATTACGGCCATGGGGAACACATGTGTAGGAC
PbrCPK2-pPR3-N	SfiI-Reversed	GGCCGAGGCGGCCCGCTACCTTTCTACTCATGCTACTT
PbrCPK3-pPR3-N	SfiI-Forward	GGCCATTACGGCCATGGGGAACAACTGCGTCGG
PbrCPK3-pPR3-N	SfiI-Reversed	GGCCGAGGCGGCCACAAACAGAGACTGCCTCCCTAA
PbrCPK7-pPR3-N	SfiI-Forward	GGCCATTACGGCCATGGGGAACACATGTGTAGGAC
PbrCPK7-pPR3-N	SfiI-Reversed	GGCCGAGGCGGCCTGCTACCTTTCTACTCATGTTACTTT
PbrCPK11-pPR3-N	SfiI-Forward	GGCCATTACGGCCATGGGAAGCTGCTTATGCAC
PbrCPK11-pPR3-N	SfiI-Reversed	GGCCGAGGCGGCCTTTTCCGTTATAGTTCGCTTGTAAC
PbrCPK18-pPR3-N	SfiI-Forward	GGCCATTACGGCCATGGGCGTTATTCACAGAGATCTCA
PbrCPK18-pPR3-N	SfiI-Reversed	GGCCGAGGCGGCCAAAAAATACATCGTCACGCCGCTT
PbrCPK20-pPR3-N	SfiI-Forward	GGCCATTACGGCCATGGGACTTTGCTTCTCCGCAG
PbrCPK20-pPR3-N	SfiI-Reversed	GGCCGAGGCGGCCCTTCCAATCTTGTACCTTCCTATCAT
PbrCPK23-pPR3-N	SfiI-Forward	GGCCATTACGGCCATGGGCAACAACTGCAGC
PbrCPK23-pPR3-N	SfiI-Reversed	GGCCGAGGCGGCCTTTTCTTCGTCTATTTGTGACCAAC
PbrCPK26-pPR3-N	SfiI-Forward	GGCCATTACGGCCATGCACCATTTGGCAGG
PbrCPK26-pPR3-N	SfiI-Reversed	GGCCGAGGCGGCCCTGTACTATTTCAAATATCAATACCACAAC
PbrCPK29-pPR3-N	SfiI-Forward	GGCCATTACGGCCATGGGGGTGATGCATAGGGA
PbrCPK29-pPR3-N	SfiI-Reversed	GGCCGAGGCGGCCCAGCCAAGTCCCCAATCCTG
PbrCPK30-pPR3-N	SfiI-Forward	GGCCATTACGGCCATGGGCAACTGTTGCTCTCAACG
PbrCPK30-pPR3-N	SfiI-Reversed	GGCCGAGGCGGCCAACAAATACATCCTCACGCCGCTTC
PbrCPK31-pPR3-N	SfiI-Forward	GGCCATTACGGCCATGGCTCCGGAGGTGCTAC
PbrCPK31-pPR3-N	SfiI-Reversed	GGCCGAGGCGGCCGTTCGCTTGTAACGATTCGTCTT
PbrCPK32-pPR3-N	SfiI-Forward	GGCCATTACGGCCATGGGTTGCTGTAGCAGCAAAC
PbrCPK32-pPR3-N	SfiI-Reversed	GGCCGAGGCGGCCAAAGAGCATTGCTGGTTGTTGAGT
PbrCPK35-pPR3-N	SfiI-Forward	GGCCATTACGGCCATGGGAAATACTTGTGTTGGA
PbrCPK35-pPR3-N	SfiI-Reversed	GGCCGAGGCGGCCCTGTACTATTTCAAATATCAATACCAC
PbrCPK39-pPR3-N	SfiI-Forward	GGCCATTACGGCCATGGGGAACTGCAACGTCTGC
PbrCPK39-pPR3-N	SfiI-Reversed	GGCCGAGGCGGCCTACGGCAATGGCTTGACCAGT
PbrCPK41-pPR3-N	SfiI-Forward	GGCCATTACGGCCATGGGCAAGTGTCTAAGCAAAAG
PbrCPK41-pPR3-N	SfiI-Reversed	GGCCGAGGCGGCCGAATAGCTTGGCCTGTGGTTGC
Primers used for Electrophysiological assays		
PbrSLAH3-PT7TS-nYFP	BglII-Forward	AGATCTATGAATTCTGGAAAACAGAAATCCC
PbrSLAH3-PT7TS-nYFP	SpeI-Reversed	ACTAGTCACCATGGCATCTCGATGCAA
PbrCPK32-PT7TS-cYFP	BglII-Forward	AGATCTATGGGTTGCTGTAGCAGCAAAC

PbrCPK32-PT7TS-cYFP	SpeI-Reversed	ACTAGTAAAGAGCATTGCTGGTTGTTGAGT
AtCPK21-PT7TS-cYFP	BglII-Forward	AGATCTATGGGTTGCTTCAGCAGTAAACA
AtCPK21-PT7TS-cYFP	SpeI-Reversed	ACTAGTATGGAATGGAAGCAGTTTCCC
AtSLAH3-PT7TS-nYFP	BglII-Forward	AGATCTATGGAGGAGAAACCAAACTATGTG
AtSLAH3-PT7TS-nYFP	SpeI-Reversed	ACTAGTTGATGAATCACTCTCTTGAGTTTTG

Table S2 CPK family genes identified in pear

Gene name	Accession number
PbrCPK1	Pbr000148.1
PbrCPK2	Pbr001308.2
PbrCPK3	Pbr001322.2
PbrCPK4	Pbr005793.1
PbrCPK5	Pbr006943.1
PbrCPK6	Pbr007868.1
PbrCPK7	Pbr010295.1
PbrCPK8	Pbr010307.1
PbrCPK9	Pbr010446.1
PbrCPK10	Pbr010447.1
PbrCPK11	Pbr011310.5
PbrCPK12	Pbr011500.1
PbrCPK13	Pbr011659.1
PbrCPK14	Pbr017027.1
PbrCPK15	Pbr017041.1
PbrCPK16	Pbr017213.1
PbrCPK17	Pbr018253.1
PbrCPK18	Pbr018323.1
PbrCPK19	Pbr021635.1
PbrCPK20	Pbr023342.1
PbrCPK21	Pbr023408.1
PbrCPK22	Pbr023960.1
PbrCPK23	Pbr024654.1
PbrCPK24	Pbr025113.1
PbrCPK25	Pbr027545.1
PbrCPK26	Pbr028710.1
PbrCPK27	Pbr028878.1
PbrCPK28	Pbr028879.1
PbrCPK29	Pbr029465.1
PbrCPK30	Pbr029596.1
PbrCPK31	Pbr030700.1
PbrCPK32	Pbr031892.1
PbrCPK33	Pbr032128.3
PbrCPK34	Pbr033297.1

PbrCPK35	Pbr033365.1
PbrCPK36	Pbr033411.1
PbrCPK37	Pbr033416.1
PbrCPK38	Pbr034839.1
PbrCPK39	Pbr036114.1
PbrCPK40	Pbr037537.1
PbrCPK41	Pbr039714.1
PbrCPK42	Pbr040137.1
PbrCPK43	Pbr042486.1
